# Be FAIR to your data

**DOI:** 10.1007/s00216-020-02526-7

**Published:** 2020-04-16

**Authors:** Dörte Solle

**Affiliations:** Leibniz Universität Hannover, Callinstr.5, 30167 Hannover, Germany

**Keywords:** Scientific data management, Lab notebooks, FAIR principles, Open access

## Abstract

Wouldn’t it be great, if experimental data were findable wherever they were? If experimental data were accessible‚ regardless of the storage place and format? If experimental data were interoperable independent of the author or its origin? If experimental data were reusable for further analysis without experimental repetition? The current state of the art of data acquisition in the laboratory is very diverse. A lot of different devices are used, analogue as well as digital ones. Usually all experimental setups and observations are summarized in a handwritten lab notebook, independently from digital or analogue sources. To change the actual and common way of laboratory data acquisition into a digital and modern one, electronic lab notebooks can be used. A challenge of science is to facilitate knowledge discovery by assisting humans and machines in their discovery of scientific data and their associated algorithms and workflows. FAIR describes a set of guiding principles to make data Findable, Accessible, Interoperable, and Reusable.

## Introduction

Wouldn’t it be great, if experimental data were findable wherever they were? Some years ago, data were stored on floppy discs or CDs. These data could get lost. Today, experimental data are distributed on various computer systems, mainly in personally defined folder structures. These data are hard to find for others who are not familiar with the folder structure. Are your data recoverable by yourself in some years or by others who might be interested in?

Wouldn’t it be great, if experimental data were accessible‚ regardless of the storage place and format? Older storage media cannot be read any more in default of the necessary devices. Some software is not used any more, or downward compatible and sometimes different programmes are used for the same purpose. Are your data still accessible in some years or by others who have not the same computer setup or software?

Wouldn’t it be great, if experimental data were interoperable independently of the author or its origin? Individual data structure and annotation make it difficult to understand and interpret foreign data. Hardly any information is given to understand the experiment or to interpret the results. The context of the data is normally not obvious. Are your data interpretable by others without any explanation?

Wouldn’t it be great, if experimental data were reusable for further analysis without experimental repetition? Without findable, accessible and interoperable experimental data, the experiment must be done again for modern data evaluation or comparison between different experiments and their results. Are your data reusable for further issues, questions or interpretation? Are your data prepared for the upcoming requirements like big data analysis or knowledge discovery by machines?

## Data acquisition today and tomorrow

**Fig. 1:**
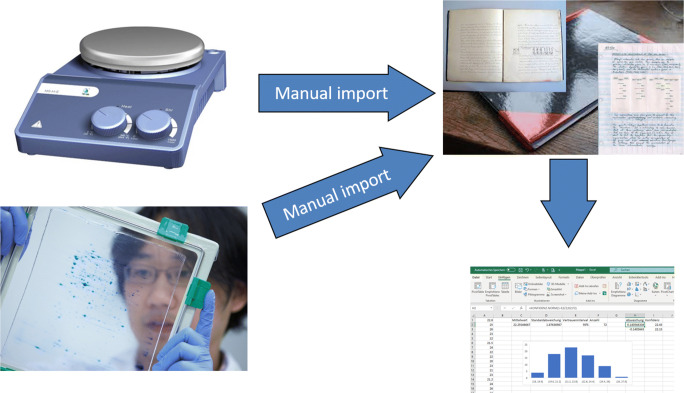
State of the art of data acquisition. The settings and observations from any devices are summarized manually in lab notebooks and transformed into individual results (pictures by Eberhard Franke and Dörte Solle)

The current state of the art of data acquisition in the laboratory is very diverse. A lot of different devices are used, analogue as well as digital ones. Images are recorded and human observations are made (see Fig. 1). Usually all experimental setups and observations are summarized in a handwritten lab notebook, independently from digital or analogue sources. Even plotted results, like chromatograms or spectra, are printed out and glued into such notebooks. The big advantage: all information and data from all the widespread sources are merged in one place. All the different formats from all the heterogeneous systems are homogenized, but unlikely in a very analogue way. This transformation is very error prone, time-consuming and goes along with a huge time delay. Additionally, these lab notebooks are only readable and reusable by the owner; in most cases, other people are not able to get any information out of it in an acceptable time.

A common way to save experimental data next to the lab notebook is on a hard disc or USB. This is a temporary solution and not save; so many people have switched to use cloud server for data storage. This simplifies data sharing with colleagues inside and outside the company and most clouds support access authorization. Without an agreement about the structure or further comments about the context of the data, cloud servers are not usable for researcher groups, especially by the exponentially growth of the data quantity. It should be questioned if this is sufficiently for digitalization and big data analysis without further compliance.

**Fig. 2:**
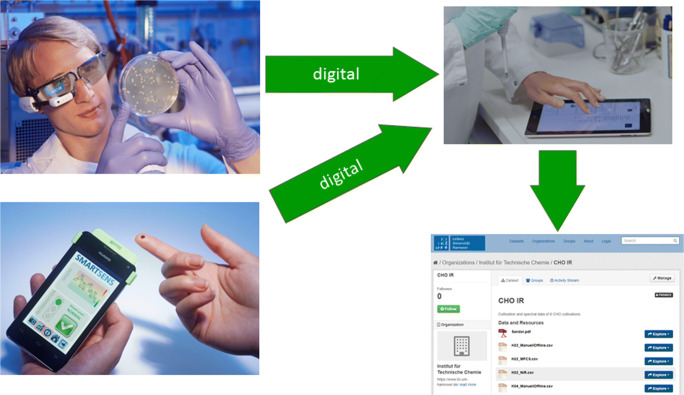
Data acquisition in the future. The settings and observation from digital devices are summarized, structured and commented in electronic lab notebook for upload to repositories (pictures by Eberhard Franke and Dörte Solle)

To change the actual and common way of laboratory data acquisition into a digital and modern one, electronic lab notebooks (ELN) can be used [1] (see Fig. 2). With ELNs you can plan experiments, document all devices setups, save digital data according to the experiment and add analogue or human observation manually. A systematic, structured or self-explaining experimental design is saved together with all necessary information about the experiment.

Different initiatives on laboratory automation, like the SiLA (Standardization in Lab Automation) consortium [2], have focused on the connection between sample processing devices and a software system for automation as described by Gauglitz [3]. Additionally, several research and developments have taken place in the area of smart laboratories [4]. The intelligent laboratory of the future is fully digitalized and uses augmented reality and modern human-computer interaction. The facilities and devices are modular with integrated functions for flexible and individual use. Till these techniques find its way into the common reality, the everyday work should be prepared for the future.

For example, by using ELNs, it is easy to share data in defined groups and the reusability of the data is not only given by the experimenter himself. To increase reusability of data, it is helpful to comment all experiments and the according data by metadata. Metadata should include descriptive information about the context, quality and condition, or characteristics of the data. Therefore, metadata are differentiated into four classes: descriptive metadata gives relevant information about the data; structural metadata shows all relationships; technical metadata provides information about the setup or analysis; and administrative metadata gives information about the author, date and confidentiality.

Necessary information in metadata are different depending on the data level. For raw data, other information should be given than for data sets, for analysed data other information is necessary than for published data. For example, row data information may include the experimental protocols, the manufacturer and sensor that created the data or the species used, but analysed data are described by workflows, algorithms, programs and so on. Up till now, there are only some common standards for metadata defined, mainly for distinct data types. In the area of analytical and bioanalytical chemistry, some examples are given for standardized data publishing. For chemical structures, certain formats are defined, together with kinetic information [5]. Mass spectroscopy information and analyses of mass spectroscopy are defined and saved together with structural information in public data bases [6]. For enzyme kinetic [7] or glycomic [8] information, a standard will be defined by the Beilstein Institute soon. All databases are specified for distinct data or focussed on certain points of view. Different databases use different file formats or metadata. This inhomogeneity is exceled by missing standards for processed data, including a description of the workflow that led to the data. More general solutions are needed, when the future requirements of research founding agencies or (open access) journals wanted to be fulfilled.

The analytical community must define these standards for their purpose: which information is obligate, which is optional. A defined format and protocol must be set up, as well as a platform to generate, read and register metadata. All analytical chemists together must go the long way to be prepared for the future requirements of science like open access, digitalization and big data analysis.

## The FAIR principle

A challenge of science is to facilitate knowledge discovery of scientific data and their associated algorithms and workflows by humans and machines. FAIR describes a set of guiding principles to make data Findable, Accessible, Interoperable, and Reusable [9, 10].

First of all, data has to be found. Data should be easy to find for both humans and computers. This is recommended by association of data with metadata. Automatic discovery of datasets is essential to enable big data analysis, and this is only possible by machine-readable metadata. The FAIR rules postulate globally unique and persistent identifiers for datasets. These identifiers will help human and machines find relevant data and reuse them in an appropriate way. Metadata should be rich, because nobody knows who will want to use the data, or for what purpose. Sometimes metadata are stored inside the data file, but this is only possible for defined row data types, like genome data. Otherwise, metadata and the datasets are separate files and have to be directly connected to each other with the globally unique and persistent identifier. This results in defined standards for data, for metadata and mainly for the association of data and metadata on different data levels.

If the data are findable, the accessibility including authentication and authorisation has to be known. For this purpose, special tools or communication methods are needed, which clearly define who can access the data and who can reuse them. This communication protocol should be free so that anyone with a computer and internet connection can access the metadata. This means not necessarily that the data themselves are free or open. It implies only that the metadata and the condition under which the associated data are accessible should be open and free. Therefore, heavily protected and private data can be FAIR data. The licensing status will become more important with automated searches by machines and the conditions under which the data can be used should be clear to machines and humans.

The next step for big data analysis is to interoperate with accessible data. Depending on the data level, information about analysis, storage and processing must be given for interpretation and a broadly applicable language for knowledge representation is needed. It is essential to use commonly used controlled vocabularies or ontologies. This vocabulary used to describe datasets needs to be documented and clearly defined. This is necessary to enrich knowledge about data and to create as many meaningful links as possible between data sets.

The ultimate goal of FAIR is the possibility to reuse data by humans and machines. To reach this, data should be well described by meaningful and rich metadata so that they can be compared for different settings. The reusability focuses on the ability to decide if the data is actually useful in a particular context. This may include information about all data levels because the data publisher does not know what the data consumer’s needs. The reuser must know where the data came from, who to cite and how the owner wanted to be confirmed. It is only possible to reuse data sets if they are similar: same type of data, data organized in a standardized way, well-established and sustainable file formats, documentation (metadata) following a common template and using common vocabulary. Community standards or best practices for data archiving and sharing have to be established for future purpose and they should be followed.

The FAIR principles represent a major challenge for research data management. This ambitious goal is supported by many different initiatives [10]. These include platforms, such as repositories, but also consortia for the development of data formats that comply with the FAIR principles. It is important for all of them that these tools are used by the researchers and that they are further developed together with them. To support this process, the DFG has launched a National Research Data Infrastructure initiative, in which subject-specific consortia are to systematically develop, sustainably secure and make available research data [11]. Some journals promote the publication of data in the course of the publication process, and even further, some funding programmes require data management plans when applying for research funds and require the publication of the data [12].

## Final consideration

To fulfil the future requirements of research founding agencies or (open access) journals, it would be a good advice for the scientific community to change the actual and common way of laboratory data acquisition into a digital and modern one. Electronic notebooks will find their way into the laboratory supported by more digital communication in the lab of future.

More digital analysis will be feasible by modern lab equipment generating much more data and the administrative work to manage this data will increase. It is recommended to follow the FAIR principles from the start in the laboratory to facilitate the reuse of the data. This requires a holistic rethinking of the scientists and an extensive reorganization of the laboratories. Hopefully data quality issues also find its way into this new lab world, because these aspects are not addressed by the FAIR principles.

Each scientific community must standardize the digital objects, which are relevant for their needs. Some formats for distinct data are designed and in use already, but for others the vocabulary, structure and format of the data is very diverse or even individual. Some manufacturers designed their own and sometimes closed formats; this is a dead-end for open access data.

We need standards for metadata. Not only for them but although for the association of data and metadata on different data levels. If raw data should be described by metadata in the data file itself, different and more complex formats are necessary and has to be defined. The infrastructure for this purpose is already given by libraries or databases. Other scientific communities clear the way for FAIR data, so the analytical community can use the established infrastructure very easily.

Let us start our journey to the internet of data for knowledge discovery by humans and machines. Be FAIR to your data and digitalize your laboratory. Make science for the future.
